# Chemical Composition of the Essential Oil of the Endemic Species *Micromeria frivaldszkyana* (Degen) Velen.

**DOI:** 10.3390/molecules24030440

**Published:** 2019-01-26

**Authors:** Valtcho D. Zheljazkov, Giuseppe Micalizzi, Ivanka Semerdjieva, Luigi Mondello

**Affiliations:** 1 Department of Crop and Soil Science, Oregon State University, 3050 SW Campus Way, 431A Crop Science Building, Corvallis, OR 97331, USA; 2 Dipartimento di Scienze Chimiche, Biologiche, Farmaceutiche ed Ambientali, University of Messina, Polo Annunziata, viale Annunziata, 98168 Messina, Italy; giumicalizzi@unime.it (G.M.); lmondello@unime.it (L.M.); 3 Department of Botany and Agrometeorology, Agricultural University, Mendeleev 12, 4000 Plovdiv, Bulgaria; v_semerdjieva@abv.bg; 4 Chromaleont s.r.l., c/o Dipartimento di Scienze Chimiche, Biologiche, Farmaceutiche ed Ambientali, University of Messina, Polo Annunziata, Viale Annunziata, 98168 Messina, Italy; 5 Unit of Food Science and Nutrition, Department of Medicine, University Campus Bio-Medico of Rome, via Alvaro del Portillo 21, 00128 Rome, Italy

**Keywords:** endemic, essential oil, medicinal plant, pulegone, menthone, neomenthol, monoterpenes, ketones

## Abstract

*Micromeria frivaldszkyana* is an endemic species found only in Bulgaria. Its essential oil (EO) composition is unknown. This study assessed the EO yield and composition of *M. frivaldszkyana* as a function of the location and of drying prior to the EO extraction. *M. frivaldszkyana* was sampled from two natural habitats, Uzana and Shipka in the Balkan Mountains; the EO was extracted via hydrodistillation and analyzed on GC/MS. The plants from the two locations had distinct EO composition. The EO content (in dried material) was 0.18% (Uzana) and 0.26% (Shipka). Monoterpene ketones were the major group of the EO constituents. Also, hydrocarbons predominated in the EO from Shipka, and alcohols predominated in the EO from Uzana. The EO from Uzana had a greater concentration of menthone (56% vs. 17% from Shipka) and neomenthol (7.8% vs. 2.4%). Conversely, the EO from Shipka had greater concentrations of pulegone (50% vs. 20% from Uzana), limonene (10.1% vs. 2.6%), and germacrene D (3.4% vs. 1.1%). Drying prior to the EO extraction altered the concentration of some constituents. This is the first report of *M. frivaldszkyana* EO yield and composition. The EO showed some similarities with the chemical profile of other Micromeria species, but overall, it has an unique chemical profile and may have distinctive applications.

## 1. Introduction

*Micromeria frivaldszkyana* (Degen) Velen. is an endemic plant species found only in Bulgaria. The genus *Micromeria* Benth. belongs to the family Lamiaceae, subfamily Nepetoideae. The number of species in the *Micromeria* genus varies in different taxonomic schemes [1]. According to Harley et al. [2], the genus comprises about 70 species, but according to another taxonomic study [3], the genus *Micromeria* includes 54 species, 32 subspecies, and 13 varieties. Plants of the genus are widely distributed from the Himalayan region to the Macaronesian Archipelago (Madeira, Cape Verde, and Canary Islands) and from the Mediterranean to South Africa and Madagascar [4]. Genetic molecular studies of the genus *Micromeria* suggest a need for a change in the taxonomic status of the genus, namely the relocation of section Pseudomelissa to Clinopodium [4].

In the official Flora Europaea and Flora of Bulgaria books, the accepted name of the genus is *Micromeria*, not *Clinopodium* [5,6]. In the Bulgarian flora, the genus is represented by four species: *Micromeria juliana* (L.) Bentham ex Reichenb., *Micromeria cristata* (Hampe) Griseb, *Micromeria dalmatica* Bentham ssp. *bulgarica* (Velen.), and *Micromeria frivaldszkyana* (Degen) Velen. [5]. There are 21 Micromeria species described in Flora Europaea, including *M. frivaldszkyana* as being a Bulgarian endemic plant [6].

The species is distributed on rocky, sunny habitats, mainly on the carbonate rock base in the Eastern and Middle Stara Planina (Balkan Mountains) [7]. The morphological features of *M. frivaldszkyana* are typical for the species from the same family, namely grassy, perennial plants; stems 15–30 cm high; strongly branched; ovoid leaves; and serrated, abaxial surface with small glands rich in EO. The plant flowers are white or pale pink, outwardly short-haired, and gathered in loose-cut inflorescences. The fruits are nutlets, up to 1 mm long, brown, and with a smooth surface. The plant is propagated with seeds [5].

Studies on this Bulgarian endemic plant are very few, and there are no refereed journal publications on the *M. frivaldszkyana* EO composition. However, there are reports on related species from the same family [8,9]. A study on the chemical composition of the EO of *M. kerneri* Murb. and *M. juliana* (L.) Benth. from Southeast Europe found that the EO of *M. kerneri* had a high concentration of oxygenated sesquiterpenes, with caryophyllene oxide as the major compound [8]. Caryophyllene oxide was also the major EO constituent of *M. juliana* from several collection sites in Montenegro except Mt. Krivošije, where piperitone oxide was the major EO constituent [8]. A study on *M. dalmatica*, *M. cristata*, and *M. juliana* in Bulgaria and Macedonia reported the essential oil profiles of the three species [9]. Another study [10] reported the EO composition of *M. cristata* and *M. juliana* collected in Serbia and Montenegro. The major EO constituents of *M. cristata* were isoborneol (11.3%), borneol (8.5%), and verbenone (8.2%), whereas the ones in the *M. juliana* EO were verbenol (11.8%), thymol (10.8%), caryophyllene oxide (10.5%), borneol (9.3%), and myrtenal (7.1%).

In a phytochemical analysis of *M. fruticosa*, a total of 215 phenolics and other compounds were tentatively identified, including some monoterpenes and sesquiterpenes [11]. A study conducted on the EO composition of *M. persica* populations in the Fars region of Iran found that the main EO constituents of the four examined populations were germacrene D, bicyclogermacrene, spathulenol, and δ-cadinene [12]. Another study [13] reported *M. fruticosa* monoterpene content and composition as a function of the development stage, day length, and day/night temperature. The main EO constituents were (+)-pulegone from very low to 80% of the oil and isomenthol from 0 to 60% depending on the leaf age, location, development stage, and temperature. *M. graeca* (L.) Bentham ex Reichenb. collected at two different locations in Attiki, Greece showed caryophyllene oxide (17.0%) and epi-α-bisabolol (12.8%) as the major constituents in the EO from one of the locations, whereas linalool (18.1%) and β-chamigrene (12.5%) were the main EO constituents from the second location [14]. The hypothesis of this study was that the EO composition of *M. frivaldszkyana* will have a unique and different profile from the EO profile of related species in the same family. Furthermore, we expected to see minor differences in EO yield and profile between the two locations although the collection sites are in the same region. In addition, we expected minor alterations in the EO composition due to drying prior to oil extraction. Since this is endemic, protected, and rare plant, we had sufficient sample only from one of the locations to test the last assumption. 

## 2. Results

### 2.1. Essential Oil (EO) Content (Yield) and Chemical Profile

The essential oil yield of dried *M. frivaldszkyana* biomass was 0.18% from Uzana and 0.26% from Shipka, however, without significant differences between them. 

#### 2.1.1. Comparison of the Chemical Families of EO Constituents as a Function of the Collection Site (Uzana and Shipka) and Drying Prior to the EO Extraction

Overall, ketones were the major group of the EO chemical constituents, and there were no differences in their concentrations due to the location or drying of the material prior to oil extraction (Table 1). Monoterpene ketones were by far the largest subgroup, with cyclic and sesquiterpene ketones being negligible. While hydrocarbons constituted the second major EO chemical group in plants from Shipka, alcohols were the second major EO chemical group in plants from Uzana. There were significant differences between the plants from Uzana and Shipka with respect to the overall content of monoterpene and sesquiterpene hydrocarbons. Drying of the plant material prior to EO extraction also resulted in a significant shift in the chemical groups of the EO constituents; hydrocarbons and alcohols were greater in the EO from the dried material than in the EO from the fresh material (Table 1). 

#### 2.1.2. Comparison of EO Constituents Between the Two Locations (Uzana vs. Shipka)

Overall, there were differences in the EO content and composition between the two locations. In addition, drying had a significant effect on EO composition, refuting our two assumptions. The EO of *M. frivaldszkyana* from Uzana had greater concentrations of menthone (56.3% vs. 18.4% in the EO from Shipka), isomenthone (0.60% vs. 0.27%), neomenthol (7.75% vs. 2.43%), menthol (0.28% vs. 0.04%), *cis*-piperitone oxide/piperitone (0.60% vs. 0.15%), neomenthyl acetate (1.08% vs. 0.21%), and eucalyptol (0.11% vs. 0.02%) (Table 1, Supplemental Figures S1–S14). In addition, camphor and *epi*-cedrol were identified in the EO samples from Uzana but not in the samples from Shipka (Table 1). 

Conversely, the EO from Shipka had higher concentrations of pulegone (50.47% vs. 20.48% in the oil from Uzana), α-Pinene (0.37% vs. 0.09%), sabinene (0.30% vs. 0.17%), β-pinene (1.30% vs. 0.36%), myrcene (0.35% vs. 0.17%), limonene (10.10% vs. 2.55%), and *trans*-isopulegone (0.64% vs. 0.43%) (Table 1). Also, the following constituents were detected in the EO from Shipka but not in the EO from Uzana: hex-(2*E*)-enal, furan, 2,5-diethyltetrahydro-, α-thujene, octan-3-ol, *p*-mentha-1(7),8-diene, phenylacetaldehyde, terpinolene, *p*-cymene, 1-octen-3-ol, acetate, 4-*trans*, 6-*cis*-allocimene, iso-isopulegol, menthofuran, *p*-cymen-8-ol, *cis*-jasmone, valerena-4,7(11)-diene, 9-*epi*-*trans*-caryophyllene, γ-muurolene, ε-amorphene, β-bisabolene, γ-cadinene, viridiflorol, salvial-4(14)-en-1-one, naphthalenol, 1,2,3,4,4a,7,8,8a-octahydro-1,6-dimethyl-4-(1-methylethyl)-, and *epi*-α-muurolol (Table 1, Supplemental Figures S1–S14).

#### 2.1.3. Essential Oil (EO) Composition from Dried vs. Fresh Material Collected at Mount Shipka

Drying had a significant effect on the EO composition of plant material collected at Mount Shipka. Drying of the plant material prior to oil extraction significantly affected the concentration of two major EO constituents; drying increased the concentration of limonene (10.1% vs. 6.9%) in the EO from the fresh material and the concentration of neomenthol (2.43% vs. 0.93%) but decreased the concentration of pulegone (50.5% vs. 61.2%) in the EO from the fresh material. In addition, the concentrations of Hex-(2*E*)-enal, sabinene, and *trans*-isopulegone were greater in the EO from the fresh material (Table 1). 

## 3. Discussion

The EO yield of dried *M. frivaldszkyana* in this study (0.18% from Uzana and 0.26% from Shipka) was comparable to the one in previous reports. Overall, the EO yield in other *Micromeria* species was found to vary significantly, from around 0.05 up to 4% [15]. For example, the EO yields in *M. cristata ssp. phrygia* collected from three sites in Turkey were 0.03–0.08% [15], the oil content of *M. cristata* and *M. juliana* collected in Serbia and Montenegro was 0.1% [10], and the EO content of *M. fruticosa* from Israel was 0.5 to 0.72% [13]. 

In this study, the main EO constituents of the endemic species *M. frivaldszkyana* collected at two locations were pulegone (20.48–61.17%), menthone (16.62–56.28%), limonene (2.55–10.10%), neomenthol (0.93–7.75%), and germacrene D (1.05–3.48%). Previous research identified three chemotypes in Micromeria species: pulegone, piperitone oxide, and carvone [1,16,17]. The *M. frivaldszkyana* plants from Shipka may belong to the pulegone chemotype based on the results from this study. However, *M. frivaldszkyana* from Uzana had menthone as the main EO constituent. Overall, *M. frivaldszkyana* from both locations had a high concentration of menthone. Therefore, we may assume the presence of a menthone chemotype (Uzana plants) and pulegone-menthone chemotype (Shipka plants). Menthone chemotype was mentioned in a recent review on other *Micromeria* species [1]. Indeed, some previous research in France identified a menthone chemotype in other species from the same family [18,19]. 

Pulegone, menthone, and limonene are monterpenes, found as constituents in the EO of a wide range of plant species [1]. Pulegone is constituent of the EO of species from the mint family such as *Nepeta cataria* and *Mentha piperita* [20], and in *Mentha pulegium*, it can constitute up to 83% of the total oil [21]. Pulegone has shown insecticidal properties and is also utilized widely as flavor and fragrance agents in perfumery, cosmetics, and aromatherapy. In 2018, the United States Food and Drug Administration (FDA) [22] reconsidered the safety and toxicity of substances and withdrew six flavoring substances (including synthetic pulegone) from the GRAS (Generally Recognized As Safe) list [22]. However, this recent ruling of 2018 did not affect natural (derived from plants) pulegone. Therefore, we anticipate greater commercial demand for the sourcing of natural pulegone. The endemic plant *M. frivaldszkyana*, a subject of this study, would have a potential as a new source for natural pulegone, if introduced into culture.

Menthone is a major EO constituent of *Mentha piperita* and other mints and geraniums and is utilized widely in perfumery and cosmetics, pharmaceutical products, e-cigarettes, and other products due to its easily identifiable soothing minty scent. Menthone is a predominant EO constituent in peppermint young growing leaves [20]. At maturation, and at senescence, however, menthone is reduced to menthol and isomenthol [20,23]. On the other hand, pulegone is reduced by pulegone reductase to produce menthone and isomenthone in peppermint [20], demonstrating the close biochemical and physiological relationships between these compounds in peppermint oil glands as a function of environmental conditions, phenological phase, or the age of the individual plant leaves, and day length [23]. Further research is needed to reveal the biosynthetic processes in *M. frivaldszkyana*. One may speculate that the identified chemotypes in Micromeria are mostly a function of growth stage, plant part, and the environment, unless there is a side-by-side comparison of these chemotypes. Limonene, the most common terpene in nature, is constituent of citrus peel EO, and it is used extensively as a flavoring agent in the food industry and various cleaning products due to its fresh citrus aroma [24]. In addition, limonene is used as a precursor for the commercial production of carvone.

There are no previous reports on the EO composition of the endemic species *M. frivaldszkyana.* However, there are reports on other species from the same family [8,9,12,13,25]. 

A study on *M. persica* from three locations in Iran [12] reported spathulenol (30.3%, 6.5%, and 10.8%), germacrene D (19.4%, 35.6%, and 22%), and bicyclogermacrene (18.9%, 15.7%, and 17.3%) as the major EO constituents. Pulegone and menthone (the major EO constituents in *M. frivaldszkyana* in this study) were not found in *M. persica* from Iran [12]. Gulluce et al. [25] reported piperitenone (50.6%), pulegone (29.2%), and isomenthone (3.92%) as the major EO constituents of *M. fruticosa* (L.) Druce ssp *serpyllifolia* (Bieb.) collected from Eastern Anatolia, Turkey. However, the *M. fruticosa* ssp. *serpyllifolia* did not have menthone (as is in this study).

In a study of *Micromeria* species from Bulgaria and Macedonia, the authors reported pulegone (35.8%), piperitenone (18.6), and *trans*-*p*-methane-3-one (15.8) as the main EO constituents of *M. dalmatica* [9]. Furthermore, caryophyllene oxide (0–14.3%), α-bisabolol (0–38.5%), geracrone (0–18.5%), and β-atlantol (0–9.9%) were the main constituents of the EO from *M. cristata* collected from three locations in Bulgaria and Macedonia. In addition, caryophyllene oxide (11.2%), spatulenol (5.6%), and *trans*-2-cren-4-ol (3.8%) were the main EO constituents of *M. juliana* from Macedonia [9]. In the same study, monoterpenes were the major group of EO constituents in the EO of *M. dalmatica* from Bulgaria and *M. cristata* from one of the locations in Bulgaria, while sesquitepenes were the major group in the *M. cristata* from the other two locations in Bulgaria and Macedonia and in *M. juliana* from Macedonia. In a study with *M. graeca* (L.) from two locations in Greece, the authors [14] reported caryophyllene oxide (17.0%) and *epi*-α-bisabolol (12.8%) as major constituents from one of the locations and linalool (18.1%) and β-chamigrene (12.5%) as the main EO constituents from the other location. *trans*-Verbenol was also relatively high in the EO of *M. graeca* from both locations. However, pulegone and menthone were not identified in the EO of *M. graeca*, whereas germacrene D constituted 0.7% and 7.5% in the EO from location 1 and 2, respectively, and limonene was 1% of the EO from one of the locations [14]. 

In a study on *M. fruticosa* in Israel, Dudai et al. [13] reported EO content (yield) variations from 0.5 to 0.72% and (+)-pulegone from 65 to 78% of the total oil as a function of day/night temperature regime and day length. Interestingly, the pulegone concentration in the leaves varied from 0% in the low base leaf pair to 71% in the tip leaf pairs, demonstrating dramatic changes within a plant and individual branch as a function of leaf age, development stage, and location. Also, the concentration of isomenthol varied from 0% in young leaves to more than 60% in older leaves, suggesting significant changes in monoterpene synthesis and accumulation during the development stages [13]. The EO content of *M. fruticosa* reported from Israel [13] was a bit higher than the one of *M. frivaldszkyana* in this study.

Kremer et al. [8] conducted a study on related species *M. kerneri* and *M. juliana* in several locations in the Balkans, including Croatia, Bosnia and Herzegovina, Montenegro, Republic of Macedonia, and Northern Greece, locations that are geographically similar and relatively close to the collection sites of *M. frivaldszkyana* in this study. The two *Micromeria* species from the above locations did not contain pulegone, menthone, or neomenthol, the major EO constituents of *M. frivaldszkyana* in this study. The limonene concentration in *M. kerneri* and *M. juliana* varied from 0% to 5.4%, while germacrene varied from 1.5% to 4.9% in the EO of the two species. 

This literature overview of the EO composition of related species, some of which were collected in the same region, confirmed our hypothesis that the EO composition of *M. frivaldszkyana* is unique and different from the EO profile of other Micromeria species in the region. Therefore, the unique EO may have novel applications. *M. frivaldszkyana*, an endemic plant, is found only on dry rocky outcrops in the Balkan Mountains of Bulgaria; it grows on a limited amount of poor soil onto the rocks and apparently has a great ecological adaptability to environments with limited nutrients and water supply. In view of its unique chemical composition, EO yield, and ecological plasticity, the plant may have a potential as a new crop and as a commercial source for high-menthone and high-pulegone EO for the flavor and fragrance industries and possibly for the food, beverage, and pharmaceutical industries. Further research is needed to evaluate the bioactivity of the plant biomass and its EO.

## 4. Materials and Methods

### 4.1. Collection of the Plant Material

An official permit (# 749/29.05.2018 of МОСВ) for collection of *Micromeria frivaldszkyana* was obtained by the authors from the Bulgarian Ministry of the Environment and Water prior to the sampling of this endemic and protected plant. *M. frivaldszkyana* was collected in August 2018 at full flowering from two locations in Bulgaria: Uzana (42°45”20.2′ N; 25°13”56′ E; 1,269 m asl) and Mount Shipka (42°44”51.5′N; 25°19”19.2′E; 1,307 m asl), both in the Natural Park Bulgarka in the Balkan Mountains (Stara Planina). The collected samples were air-dried at room temperature for a couple of weeks until a constant weight. Voucher specimens of *Micromeria frivaldszkyana* (small branches with leaves and flowers) were deposited at the Herbarium of the Agricultural University, Plovdiv, Bulgaria (SOA) [26].

### 4.2. Essential Oil (EO) Extraction of the M. frivaldszkyana Biomass Samples

Subsamples from the whole above ground plant parts of *Micromeria frivaldszkyana* (stems, leaves, and inflorescences) were submitted to hydrodistillation for EO extraction. The EO was extracted in 2 L hydro-distillation units (Laborbio Ltd., Sofia, Bulgaria, laborbio.com); each distillation was done in two replicates. The oil was measured by volume, transferred in 2 mL vials and placed in a freezer. Afterwards, the oil samples were separated from the remaining water, measured on an analytical scale, and kept in a freezer until they were analyzed. Six samples of *Micromeria* (2 locations × 2 replicates extracted from dried material and one sample from Mount Shipka (Balkan Mountains, Bulgaria) in two replicates was extracted fresh). Samples were stored at −3 °C. Before analysis, the samples were defrosted at room temperature.

### 4.3. Samples, Sample Preparation, and Gas-Chromatography/MS (Table S1 and Figures S1–S14)

All samples (10 μL) were dissolved in 990 μL of n-hexane and injected on the GC-MS and GC-FID systems. 

#### 4.3.1. GC-MS Analysis

The essential oil (EO) analyses were carried out on a GCMS-QP2020 (Shimadzu, Milan, Italy) equipped with a split–splitless injector, an AOC-20i autosampler, and a quadrupole MS detector. MS parameters were as follows: mass range 40–550 amu, scan speed 3333 amu/s, ion source temperature 220 °C, and interface temperature 250 °C. A SLB-5ms 30 m × 0.25 mm id × 0.25 μm film thickness column (Merck Life Science (Merck KGaA, Darmstadt, Germany)) (silphenylene polymer virtually equivalent in polarity to poly (5% diphenyl/95% dimethyl siloxane phase)) was used for the characterization of the volatile fraction. The column operated under a programmed temperature of 50 °C to 280 °C (5 min) at 3.0 °C/min. Injection volumes and mode were 1.0 μL; the split ratio was 10:1. Helium was used as a gas carrier at a constant linear velocity of 30 cm/s. The *GCMS solution* software (version 4.41 Shimadzu, Milan, Italy) was used for data collection and handling. A homologous series of *n*-alkanes (C7-C30 Saturated Alkanes, Merck Life Science, Darmstadt, Germany) standard solution has been used for Linear Retention Indices (LRIs) calculation that supported the identification of analytes on the SLB-5ms column. The peaks assignment was carried out based on a double filter, namely the MS similarity spectra (over 85%) and a LRIs ± 5 compared to the values reported in the spectral library. For mass spectral identification, Shimadzu *FFNSC 3.01* was mainly used.

#### 4.3.2. GC-FID Analysis

Quantitative analyses were carried out on a GC-2010 Plus (Shimadzu, Milan, Italy) equipped with a split–splitless injector (280 °C), an AOC-20i autosampler, and a flame ionization detector (FID). A SLB-5 ms 30 m × 0.25 mm id × 0.25 μm film thickness column (Merck Life Science) operated under the programmed temperature of 50 °C to 280 °C (5 min) at 3.0 °C/min. Injection volume and mode were 1.0 μL; the split ratio was 10:1. Helium was used as the carrier at a constant linear velocity of 30 cm/s and a pressure of 99.5 KPa. The FID temperature was set at 280 °C (sampling rate 60 ms), and gas flows were 40 mL/min for hydrogen, 30 mL/min for make up (nitrogen), and 400 mL/min for air. Data were processed through the *LabSolution* software (version 5.92 Shimadzu, Milan, Italy). Quantification was performed using the GC-FID data. Each sample was analyzed for three consecutive runs for a major precision of data.

### 4.4. Statistical Analysis

All data was analyzed with the one-way analysis of variance (ANOVA) to reveal significant differences between the means of each of the essential oil constituents from the two locations and between dried and fresh material using JMP Pro program (SAS Institute, Cary, North Carolina, United States). When F < 0.05, the means were compared using Tukey HSD and letter groupings were generated by the system. 

## Figures and Tables

**Table 1:** The identification of the volatile fraction in *Micromeria frivaldszkyana* essential oils by using LRI (Linear Retention Index). LRI lib are values reported in FFNSC 3.01 library; LRI exp are obtained experimentally on SLB-5ms column. % MS Sim. represents the similarity between experimental and library spectra. The quantification of flavour and fragrance compounds in *Micromeria frivaldszkyana* essential oils by GC-FID % area values are the average of three repetitions.

ID	Compounds	% MS Sim.	LRI Exp	LRI Lib	Uzana Dry	Shipka Dry	Shipka Fresh
1	Hex-(2*E*)-enal	94	850	850		0.09 *	0.02 b
2	Furan, 2,5-diethyltetrahydro-	91	897	896		0.01	0.01
3	α-Thujene	92	925	927		0.01	0.02
4	α-Pinene	95	934	933	0.09 b	0.37 a	0.51 a
5	Sabinene	95	973	972	0.17 c	0.30 b	0.36 a
6	β-Pinene	93	979	978	0.36 b	1.30 a	1.66 a
7	Octan-3-one	94	984	986		0.07 a	0.04 a
8	Myrcene	95	989	991	0.17 b	0.35 a	0.41 a
9	Octan-3-ol	96	997	999		0.08 a	0.08 a
10	*p*-Mentha-1(7),8-diene	95	1005	1004		0.03 a	0.04 a
11	*p*-Cymene	93	1025	1025	0.01 a	0.04 a	0.02 a
12	Limonene	94	1030	1030	2.55 c	10.10 a	6.93 b
13	Eucalyptol	96	1033	1032	0.11 a	0.02 b	0.02 b
14	*cis*-β-Ocimene	90	1035	1035	0.07 b	0.22 ab	0.30 a
15	Phenylacetaldehyde	92	1044	1044		tr	0.01
16	*trans*-β-Ocimene	94	1046	1046	0.03 b	0.12 ab	0.15 a
17	γ-Terpinene	92	1059	1058		0.03 a	0.01 a
18	*cis*-Sabinene hydrate	88	1071	1069	0.07	0.02	0.02
19	Terpinolene	95	1087	1086		0.02	0.02
20	*p*-Cymenene	94	1092	1093		0.01	0.01
21	Linalool	93	1100	1101	0.04	0.01	0.01
22	*n*-Nonanal	91	1105	1107	0.02	0.04	0.03
23	1-Octen-3-ol, acetate	95	1107	1109		0.01	0.03
24	*trans*-*p*-Mentha-2,8-dien-1-ol	87	1123	1122	0.03	0.02	0.02
25	4-*trans*, 6-*cis*-Allocimene	90	1129	1128		0.03	0.04
26	*cis*-*p*-Mentha-2,8-dien-1-ol	93	1139	1138	0.02	0.02	0.02
27	Camphor	96	1150	1149	1.14		
28	*p*-Menth-3-en-8-ol	88	1153	1149	0.03	0.07	0.04
29	Menthone	94	1160	1158	56.28 a	18.42 b	16.62 b
30	iso-Isopulegol	95	1162	1160		0.02	0.01
31	Menthofuran	91	1165	1164		0.02	0.04
32	Isomenthone	94	1166	1166	0.60 a	0.27 b	0.24 b
33	Neomenthol	95	1172	1170	7.75 a	2.43 b	0.93 c
34	Borneol	94	1175	1173	0.93	0.04	0.01
35	*trans*-Isopulegone	93	1177	1175	0.43 c	0.64 b	0.78 c
36	Menthol	95	1180	1184	0.28 a	0.04 b	0.03 b
37	Terpinen-4-ol	92	1182	1184	0.15		
38	*p*-Cymene-8-ol	92	1192	1189		0.06 a	0.04 a
39	α-Terpineol	94	1198	1195	0.05	0.09	0.12
40	*trans*-Carveol	92	1224	1223		0.01	
41	*cis*-3-Hexenyl isovalerate	95	1237	1235		0.01	
42	Pulegone	93	1244	1241	20.48 c	50.47 b	61.17 a
43	Carvone	95	1248	1246	0.06	0.03	0.03
44	*cis*-Piperitone oxide	90	1257	1255	0.60 a	0.15 b	0.50 ab
45	Piperitone *^A^	93	1261	1267			
46	Neomenthyl acetate	96	1273	1272	1.08 a	0.21 b	0.22 b
47	Bornyl acetate	96	1286	1285	0.09		
48	Thymol	97	1292	1293		2.81 a	0.05 a
49	Carvacrol	94	1300	1300		0.34	0.01
50	Bicycloelemene	94	1334	1338		0.34 a	0.27 a
51	Piperitenone	95	1341	1343	0.12 b	0.18 b	0.40 a
52	Piperitenone oxide	92	1365	1372	0.15 b	0.33 ab	0.80 a
53	α-Copaene	98	1378	1375	0.15	0.14	0.11
54	*trans*-β-Damascenone	90	1381	1379		0.01	
55	β-Bourbonene	96	1386	1384	0.79 a	0.48 ab	0.30 b
56	1,5-Di-*epi*-β-bourbonene	88	1389	1390	0.26 a	0.30 a	0.27 a
57	β-Elemene *^B^	92	1391	1390			
58	*cis*-Jasmone	93	1395	1394		0.02	0.01
59	β-Ylangene	88	1421	1422	0.17 a	0.24 a	0.22 a
60	*trans*-Caryophyllene	95	1423	1424	0.07 a	0.13 a	0.04 a
61	β-Copaene	97	1433	1433	0.15 a	0.18 a	0.15 a
62	Isogermacrene D	94	1448	1447	0.14 a	0.12 a	0.09 a
63	Valerena-4,7(11)-diene	90	1455	1455		0.08	0.07
64	9-*epi*-*trans*-Caryophyllene	91	1464	1464		0.07	0.03
65	γ-Muurolene	94	1479	1478		0.07	0.01
66	Germacrene D	91	1485	1480	1.05 b	3.40 a	3.48 a
67	Bicyclogermacrene	96	1499	1497	0.07 a	0.82 a	0.62 a
68	ε-Amorphene	94	1503	1502		0.04	0.04
69	β-Bisabolene	95	1509	1508		0.33	
70	γ-Cadinene	95	1516	1512		0.04	0.01
71	δ-Cadinene	97	1521	1518	0.03 b	0.17 a	0.11 ab
72	Spathulenol	92	1581	1576	0.72 a	0.80 a	0.21 a
73	Viridiflorol	93	1594	1594		0.03	0.01
74	Salvial-4(14)-en-1-one	90	1597	1599		0.10	0.07
75	*epi*-Cedrol	93	1617	1621	0.30		
76	1-Naphthalenol, 1,2,3,4,4a,7,8,8a-octahydro-1,6-dimethyl-4-(1-methylethyl)-	93	1641	1646		0.03	0.02
77	*epi*-α-Muurolol	94	1648	1645		0.03	0.02
78	Cadin-4-en-10-ol	94	1660	1659	0.15	0.09	0.06
79	**Not identified ^b^**				2.10	2.11	1.07
	Hydrocarbons				6.29 c	19.84	16.26 b
	*Monoterpene*				3.44 c	12.91 b	10.46 b
	*Sesquiterpene*				2.85 b	6.93 a	5.80 a
	Aldehydes				0.02 b	0.13 a	0.06 ab
	*Aliphatic*				0.02 b	0.13 a	0.05 b
	*Aromatic*					tr	0.01
	Ketones				79.69 a	70.34 a	79.84 a
	*Aliphatic*					0.07 a	0.04 a
	*Cyclic*					0.02	0.01
	*Monoterpene*				79.69 a	70.15 a	79.72 a
	*Sesquiterpene*					0.10	0.07
	Alcohols				10.48 a	7.01 a	1.68 b
	*Aliphatic*					0.08 a	0.08 a
	*Monoterpene*				9.32 a	5.96 a	1.29
	*Sesquiterpene*				1.16	0.97	0.31
	Esters				1.16 a	0.22 b	0.22 b
	*Aliphatic*					0.01	
	*Monoterpene*				1.16 a	0.21 b	0.22 b
	Ethers				0.00	0.03 a	0.05 a
	Oxides				0.26 b	0.36 b	0.85 a

* Means followed by the same letter within a row are not significantly different at the 5% level of significance. *^A^ coelution between Piperitone oxide <cis-> and Piperitone on SLB-5ms column. *^B^ coelution between 1,5-di-epi-beta-bourbonene and Elemene <β-> on SLB-5ms column. ^b^ the sum of the not identified components.

## Supplementary Materials

The following are available online at http://www.mdpi.com/1420-3049/24/3/440/s1, Figures S1–S14. GC-MS chromatograms of the samples; Table S1: Identification of volatile fraction in *Micromeria frivaldszkyana* essential oils by using LRI (Linear Retention Index). LRI lib are values reported in FFNSC 3.01 library; LRI exp are obtained experimentally on SLB-5ms column. % MS Sim. represents the similarity between experimental and library spectra.
