# Supplementary material for: Chemical Composition of the Essential Oil of the Endemic Species Micromeria frivaldszkyana (Degen) Velen
Source: Molecules. 2019 Jan 26;24(3):440. doi: 10.3390/molecules24030440 (PMC6385347; doi:10.3390/molecules24030440)
Supplement: Supplementary file 1 [file molecules-24-00440-s001.pdf]

Table S1: *Identification of volatile fraction in Micromeria essential oils by using LRI (Linear Retention Index). LRI lib are values reported in FFNSC 3.01 library; LRI exp are obtained experimentally on SLB-5ms column. % MS Sim. represents the similarity between experimental and library spectra.*

ID	Compounds	% MS Sim.	LRI exp	LRI lib
1	Hex-(2E)-enal	94	850	850
2	Furan, 2,5-diethyltetrahydro-	91	897	896
3	α-Thujene	92	925	927
4	α-Pinene	95	934	933
5	Sabinene	95	973	972
6	β-Pinene	93	979	978
7	Octan-3-one	94	984	986
8	Myrcene	95	989	991
9	Octan-3-ol	96	997	999
10	*p*-Mentha-1(7),8-diene	95	1005	1004
11	*p*-Cymene	93	1025	1025
12	Limonene	94	1030	1030
13	Eucalyptol	96	1033	1032
14	*cis*-β-Ocimene	90	1035	1035
15	Phenylacetaldehyde	92	1044	1044
16	*trans*-β-Ocimene	94	1046	1046
17	γ-Terpinene	92	1059	1058
18	*cis*-Sabinene hydrate	88	1071	1069
19	Terpinolene	95	1087	1086
20	*p*-Cymenene	94	1092	1093
21	Linalool	93	1100	1101
22	*n*-Nonanal	91	1105	1107
23	1-Octen-3-ol, acetate	95	1107	1109
24	*trans*-*p*-Mentha-2,8-dien-1-ol	87	1123	1122
25	4-*trans*, 6-*cis*-Allocimene	90	1129	1128
26	*cis*-*p*-Mentha-2,8-dien-1-ol	93	1139	1138
27	Camphor	96	1150	1149
28	*p*-Menth-3-en-8-ol	88	1153	1149
29	Menthone	94	1160	1158
30	iso-Isopulegol	95	1162	1160
31	Menthofuran	91	1165	1164
32	Isomenthone	94	1166	1166
33	Neomenthol	95	1172	1170
34	Borneol	94	1175	1173
35	*trans*-Isopulegone	93	1177	1175 *
36	Menthol	95	1180	1184
37	Terpinen-4-ol	92	1182	1184
38	*p*-Cymene-8-ol	92	1192	1189
39	α-Terpineol	94	1198	1195
40	*trans*-Carveol	92	1224	1223
41	*cis*-3-Hexenyl isovalerate	95	1237	1235
42	Pulegone	93	1244	1241
43	Carvone	95	1248	1246

44	*cis*-Piperitone oxide	90	1257	1255
45	Piperitone *[A]	93	1261	1267
46	Neomenthyl acetate	96	1273	1272
47	Bornyl acetate	96	1286	1285
48	Thymol	97	1292	1293
49	Carvacrol	94	1300	1300
50	Bicycloelemene	94	1334	1338
51	Piperitenone	95	1341	1343
52	Piperitenone oxide	92	1365	1372
53	α-Copaene	98	1378	1375
54	*trans*-β-Damascenone	90	1381	1379
55	β-Bourbonene	96	1386	1384
56	1,5-Di-*epi*-β-bourbonene	88	1389	1390
57	β-Elemene *[B]	92	1391	1390
58	*cis*-Jasmone	93	1395	1394
59	β-Ylangene	88	1421	1422
60	*trans*-Caryophyllene	95	1423	1424
61	β-Copaene	97	1433	1433
62	Isogermacrene D	94	1448	1447
63	Valerena-4,7(11)-diene	90	1455	1455
64	9-*epi*-*trans*-Caryophyllene	91	1464	1464
65	γ-Muurolene	94	1479	1478
66	Germacrene D	91	1485	1480
67	Bicyclogermacrene	96	1499	1497
68	ε-Amorphene	94	1503	1502
69	β-Bisabolene	95	1509	1508
70	γ-Cadinene	95	1516	1512
71	δ-Cadinene	97	1521	1518
72	Spathulenol	92	1581	1576
73	Viridiflorol	93	1594	1594
74	Salvial-4(14)-en-1-one	90	1597	1599
75	*epi*-Cedrol	93	1617	1621
76	1-Naphthalenol, 1,2,3,4,4a,7,8,8a-octahydro-1,6-dimethyl-4-(1-methylethyl)-	93	1641	1646
77	*epi*-α-Muurolol	94	1648	1645
78	Cadin-4-en-10-ol	94	1660	1659

molecules
MDPI

Supplemental Figures S1-S14: *GC-MS chromatograms of individual essential oil samples.*

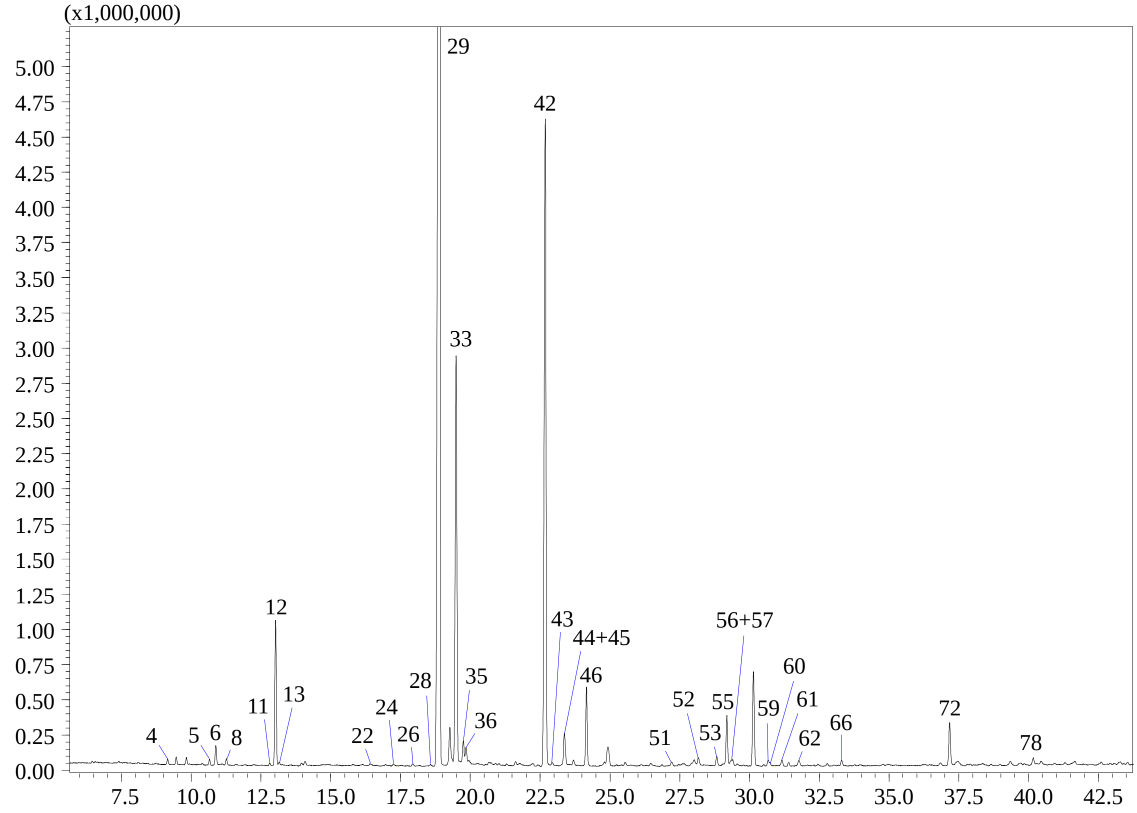

Figure 1. *GC-MS chromatogram relative to the analysis of "Micromeria – Uzana rep 1 dried"essential oil sampleon SLB-5ms column.*

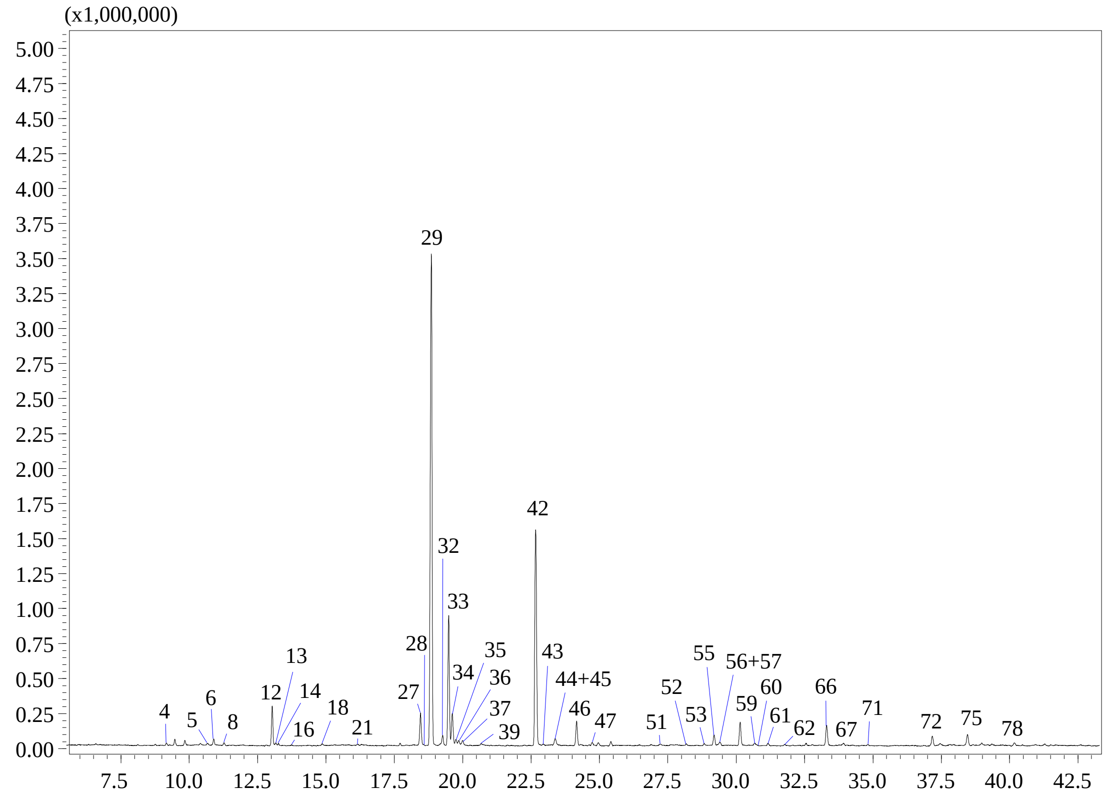

Figure 2. *GC-MS chromatogram relative to the analysis of "Micromeria – Uzana rep 2 dried"essential oil sample on SLB-5ms column.*

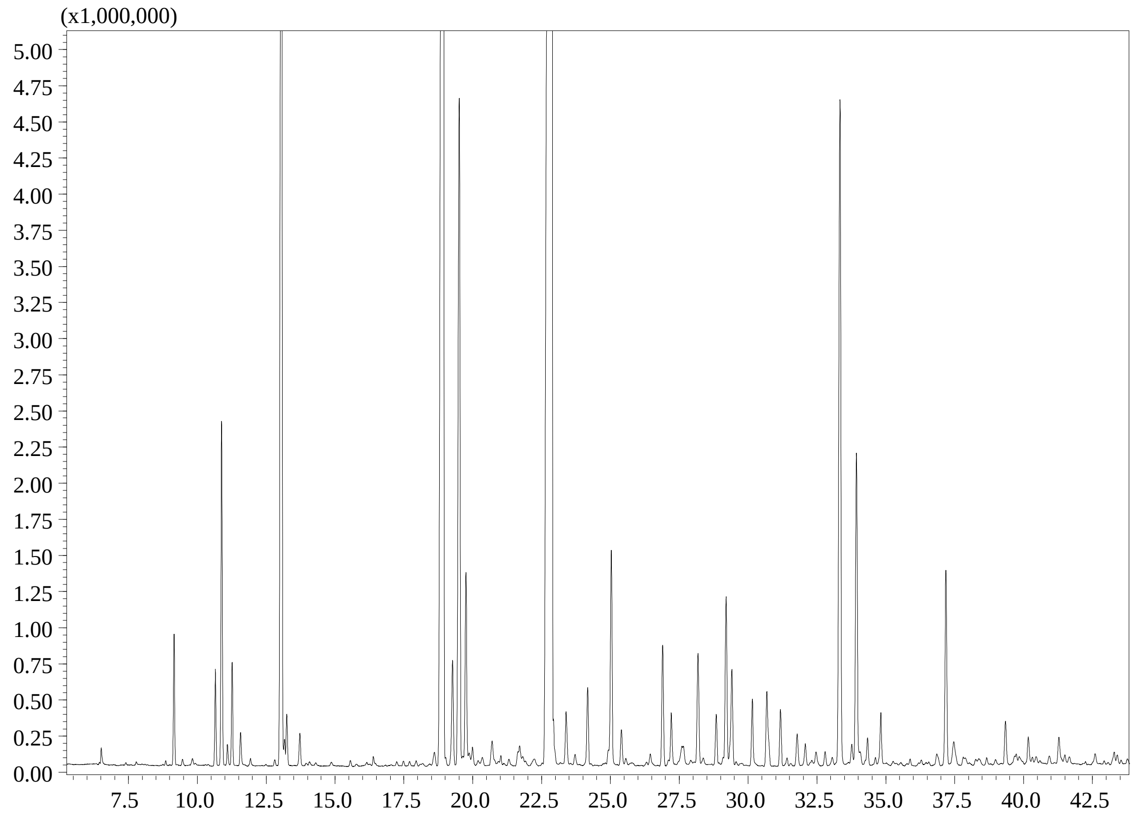

Figure 3. *GC-MS chromatogram relative to the analysis of "Micromeria – Shipka rep 1 dried"essential oil sample on SLB-5ms column.*

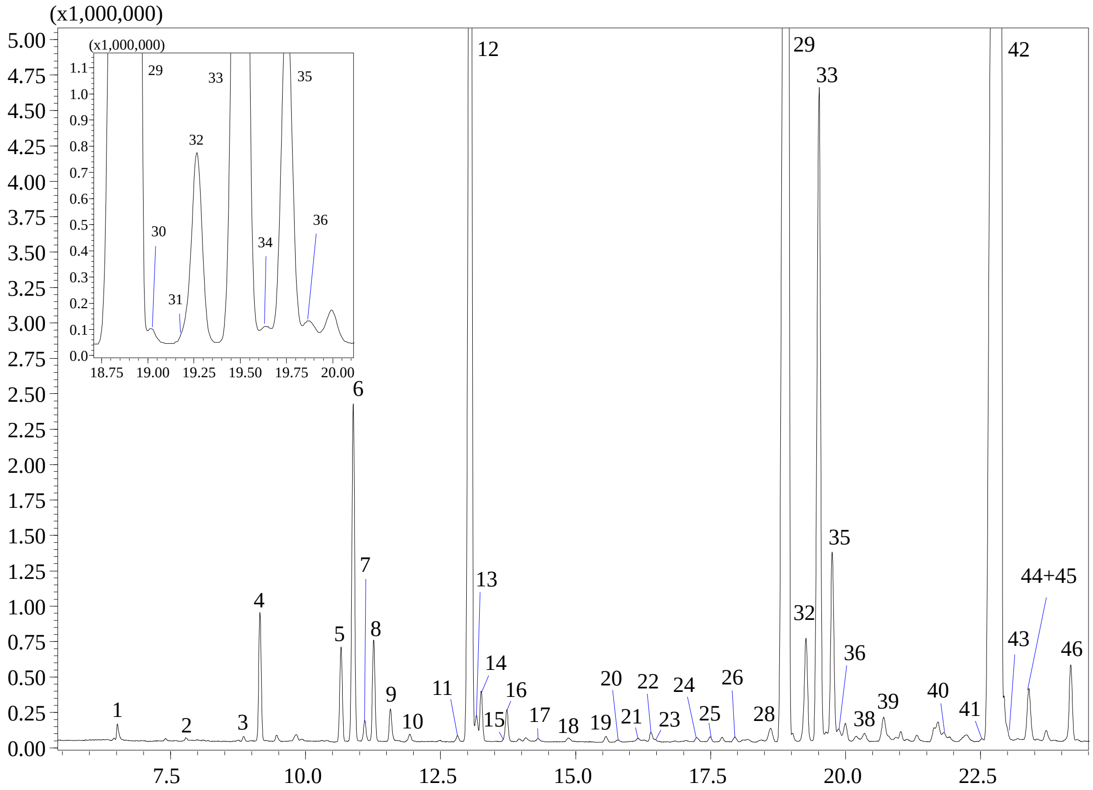

Figure 4. *Expansion (5-25min) of GCMS chromatogram relative to the analysis of "Micromeria – Shipka rep 1 dried" essential oil sample.*

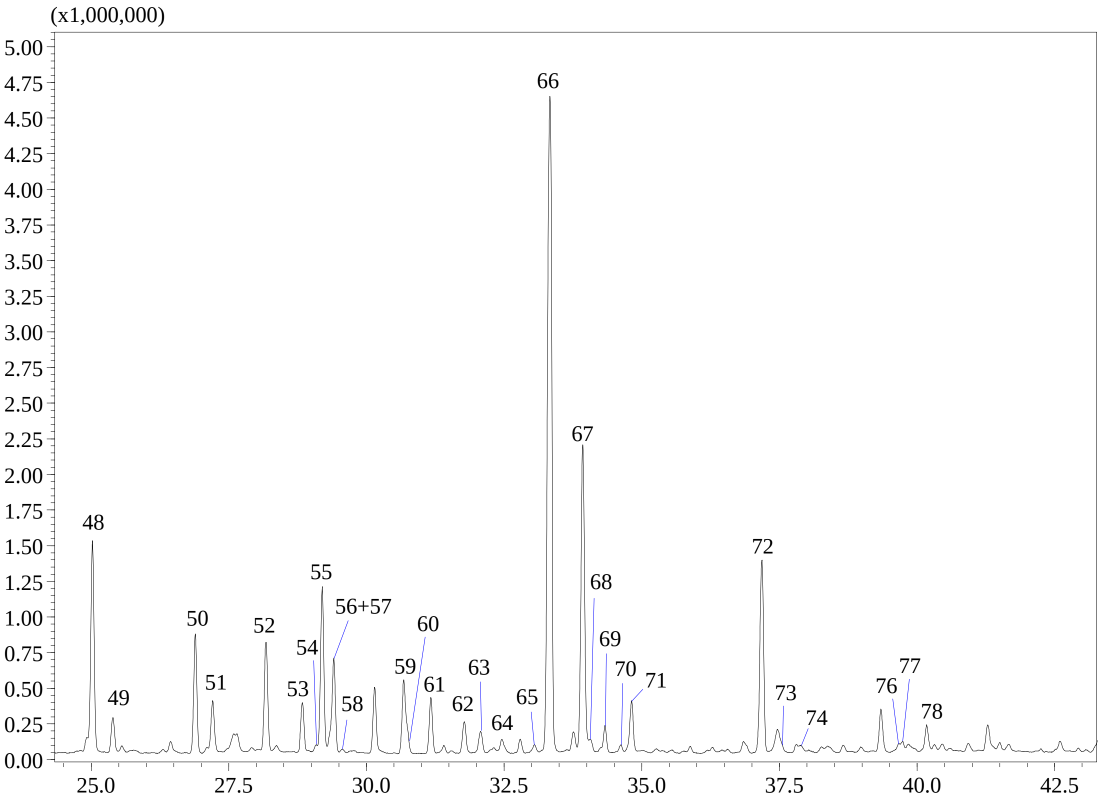

Figure 5. *Expansion (25-43min) of GCMS chromatogram relative to the analysis of "Micromeria – Shipka rep 1 dried" essential oil sample.*

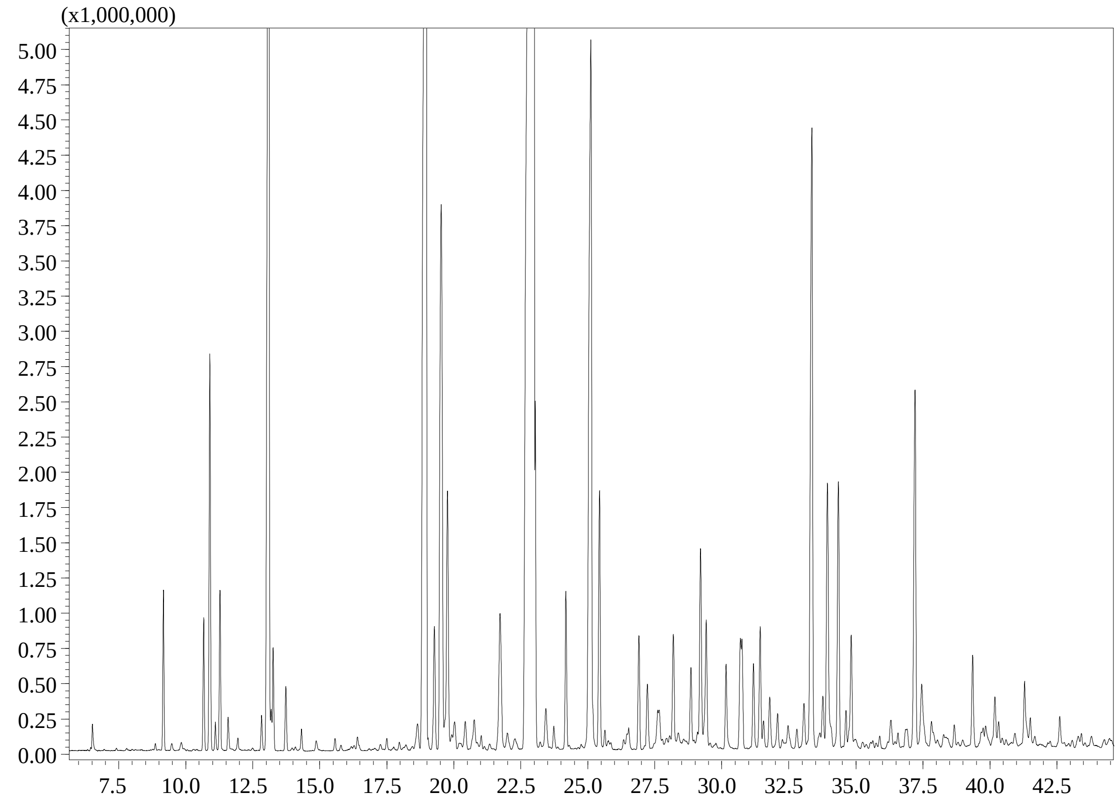

Figure 6. *GC-MS chromatogram relative to the analysis of "Micromeria – Shipka rep 2 dried"essential oil sample on SLB-5ms column.*

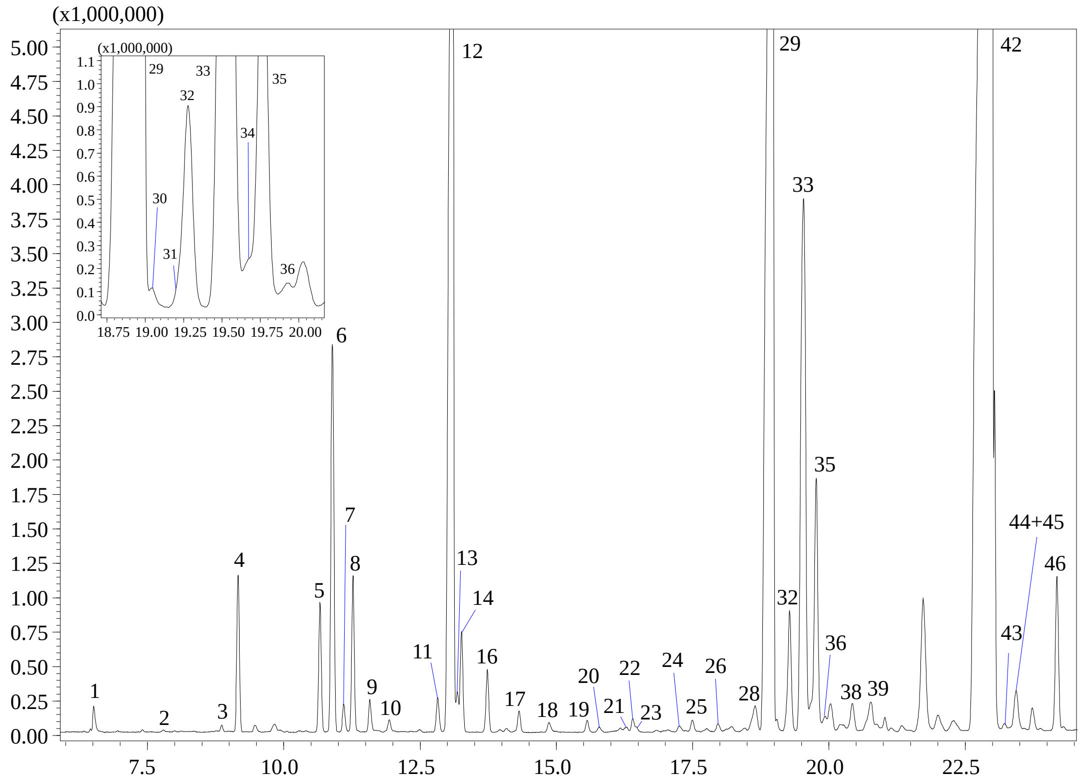

Figure 7. *Expansion (5-25min) of GCMS chromatogram relative to the analysis of "Micromeria – Shipka rep 2 dried" essential oil sample.*

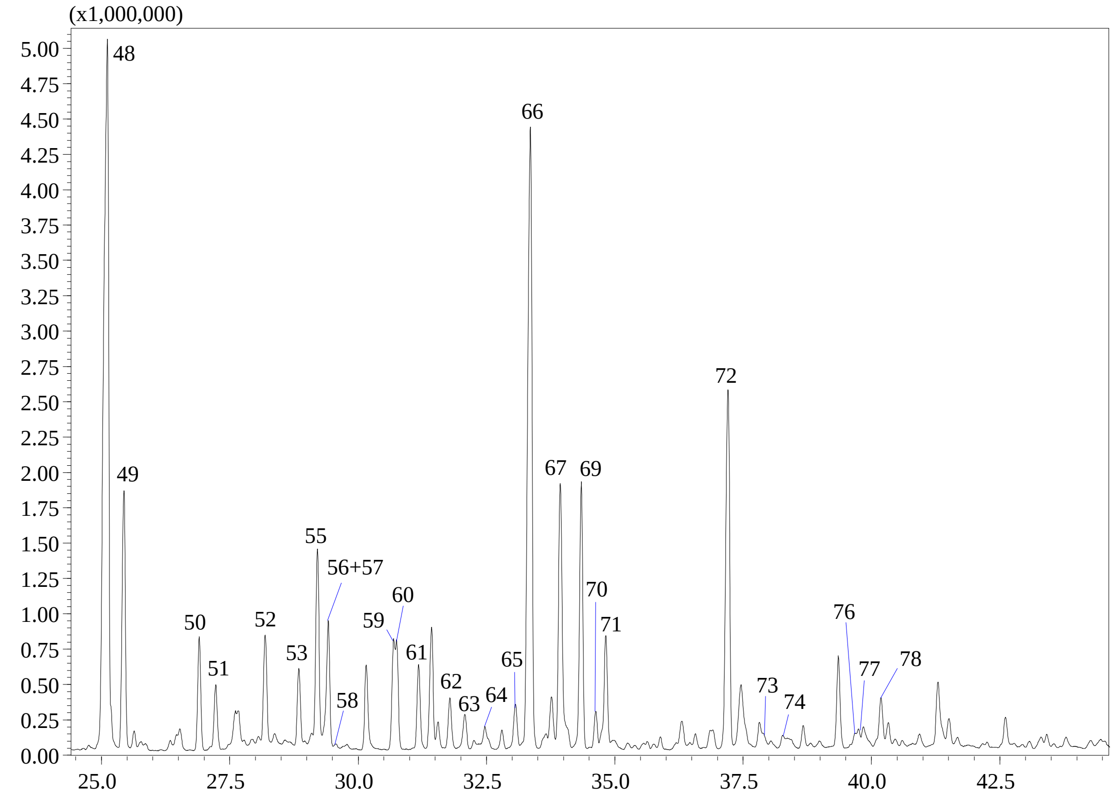

Figure 8. *Expansion (25-43min) of GCMS chromatogram relative to the analysis of "Micromeria – Shipka rep 2 dried" essential oil sample.*

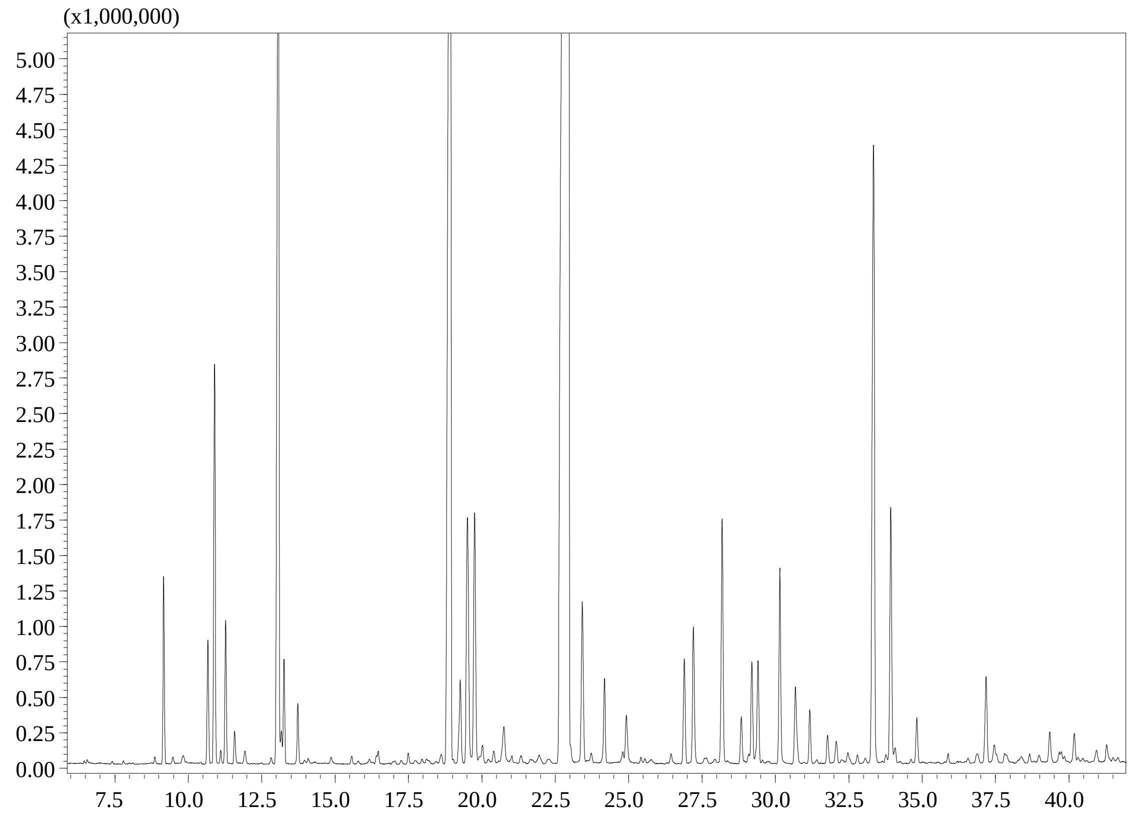

Figure 9. *GC-MS chromatogram relative to the analysis of "Micromeria – "Shipka rep 1 fresh" essential oil sample on SLB-5ms column.*

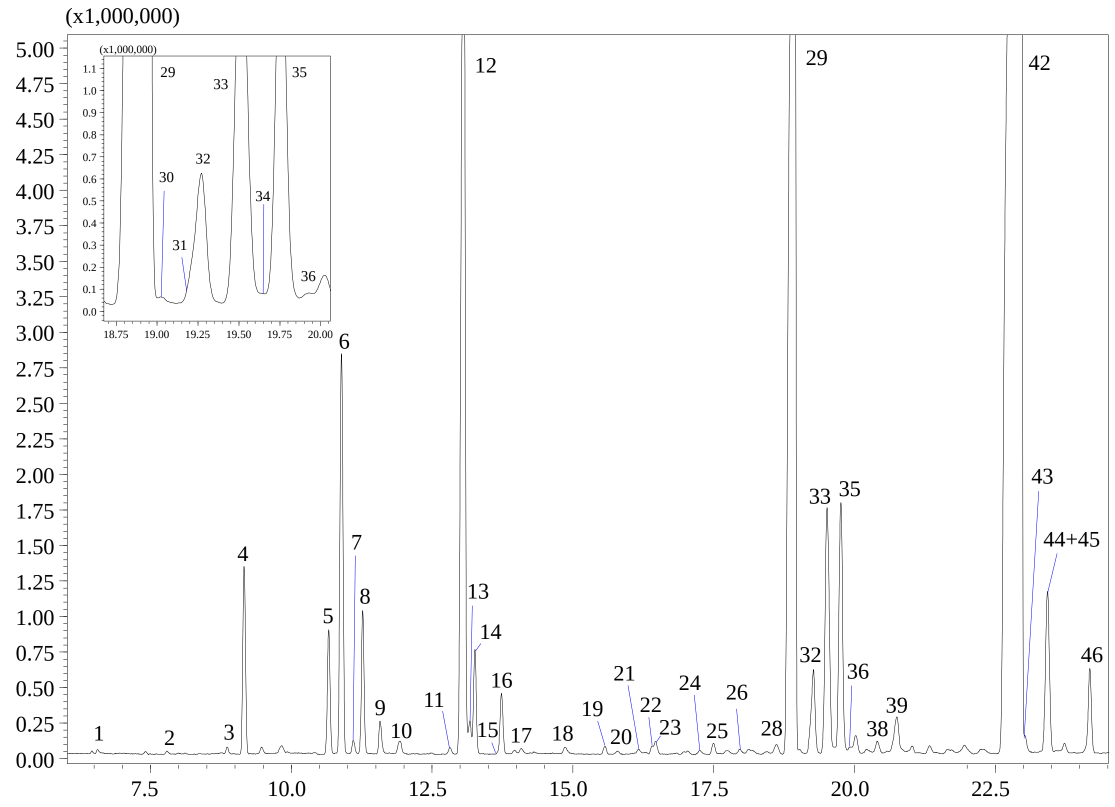

Figure 10. *Expansion (5-25min) of GCMS chromatogram relative to the analysis of "Micromeria – Shipka rep 1 fresh" essential oil sample.*

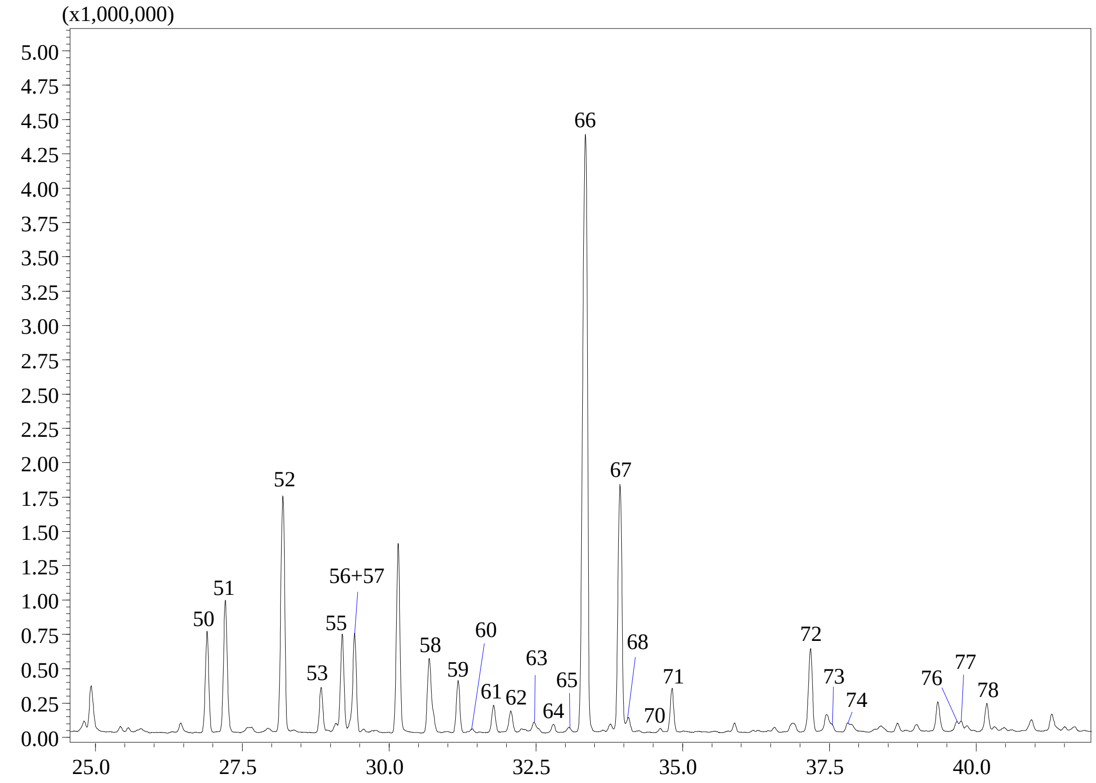

Figure 11. *Expansion (25-43min) of GCMS chromatogram relative to the analysis of "Micromeria – Shipka rep 1 fresh" essential oil sample.*

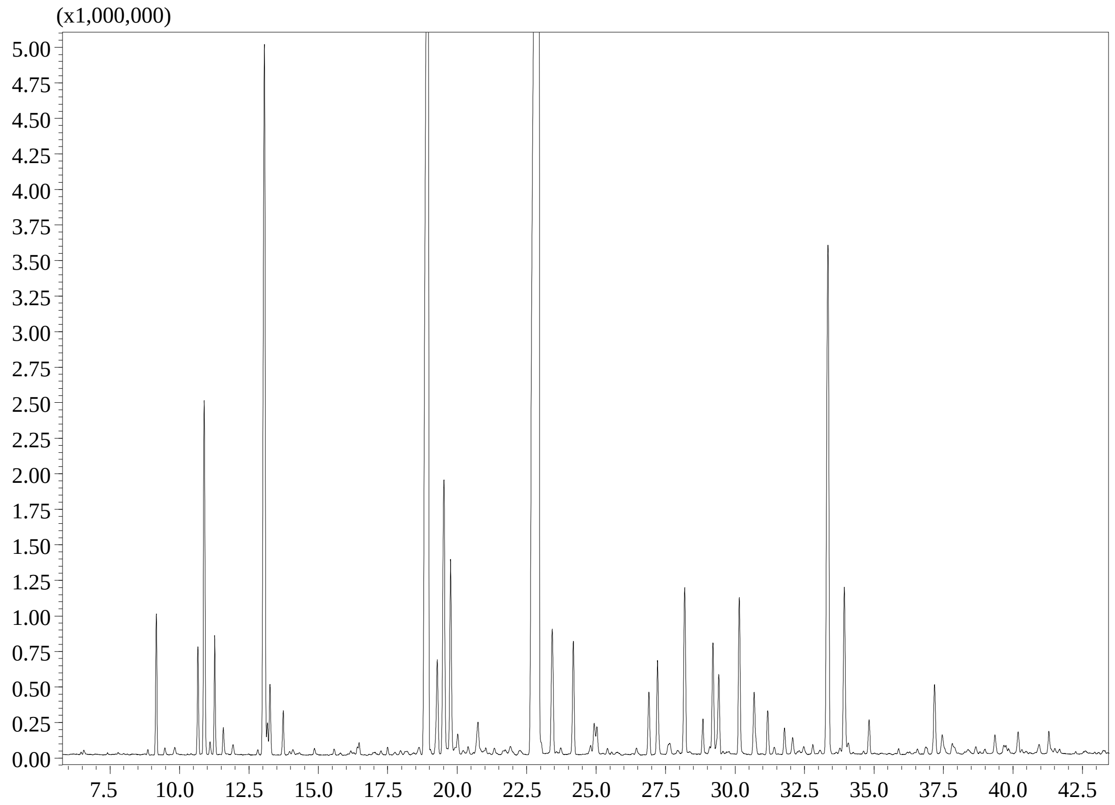

Figure 12. *GC-MS chromatogram relative to the analysis of "Micromeria –Shipka rep 2 fresh" essential oil sample on SLB-5ms column.*

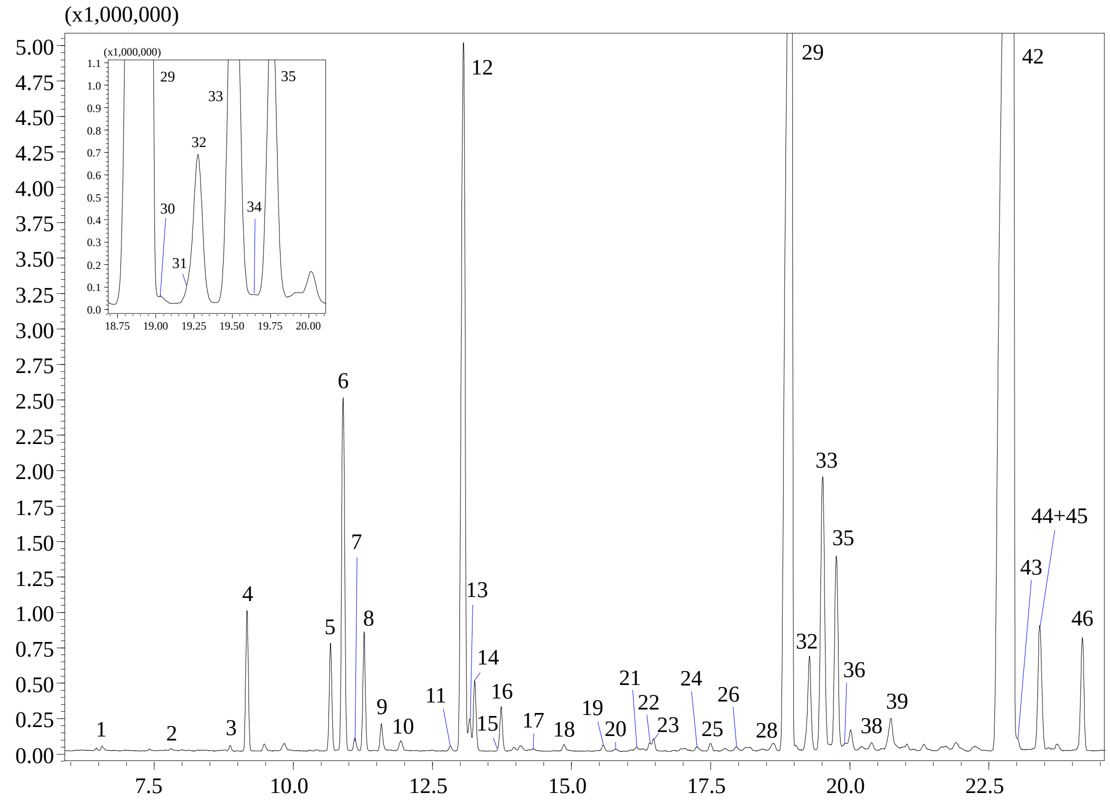

Figure 13. *Expansion (5-25min) of GCMS chromatogram relative to the analysis of "Micromeria – Shipka rep 2 fresh" essential oil sample.*

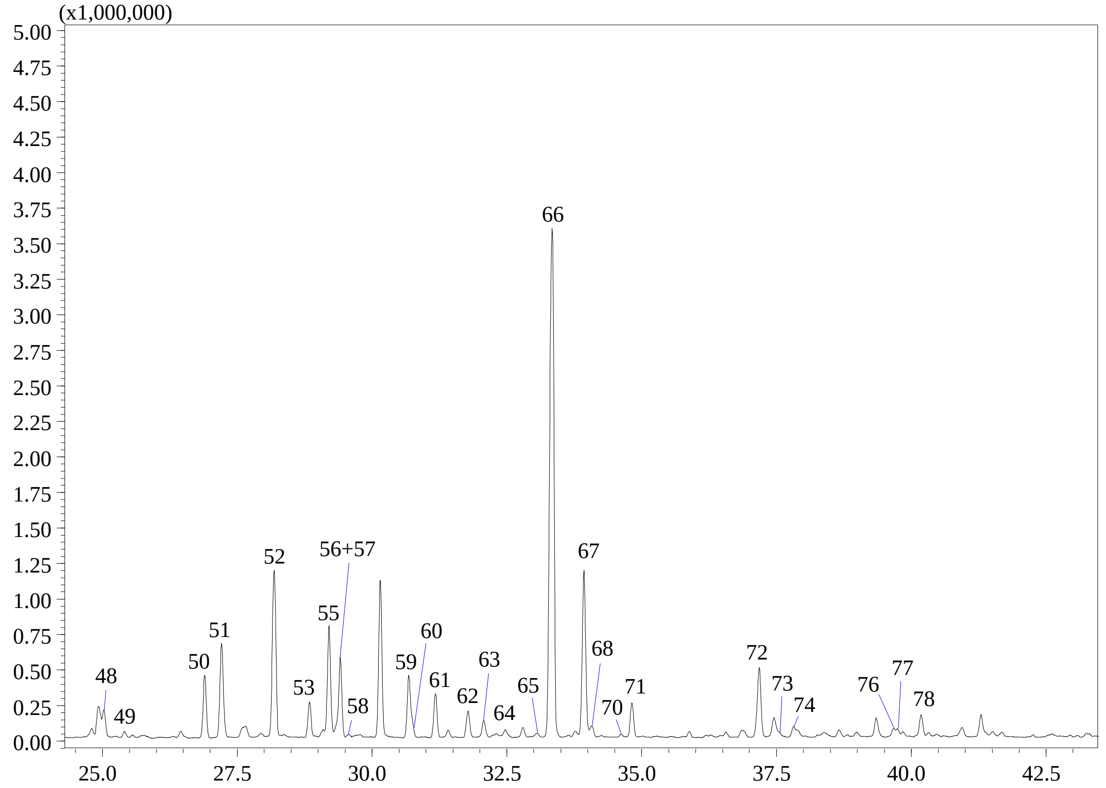

Figure 14. *Expansion (25-43min) of GCMS chromatogram relative to the analysis of "Micromeria –Shipka rep 2 fresh" essential oil sample.*
